# Supplementary material for: Clinical significance of hypoalbuminemia in patients with scrub typhus complicated by acute kidney injury
Source: PLoS One. 2021 Feb 25;16(2):e0247584. doi: 10.1371/journal.pone.0247584 (PMC7906298; doi:10.1371/journal.pone.0247584)
Supplement: Attachment — Submitted filename: Response to reviewer comments.docx [file pone.0247584.s002.docx]

**Dear Prof. Bhagwan Dass**

Academic Editor, PLOS ONE

Thank you very much for the evaluation of our manuscript. We are returning a revised manuscript which incorporates many of the suggestions made by one reviewer. A response to the referees΄ suggestions has been listed one by one, and an index of the changes has been included. We hope that the comments of the referees are adequately addressed in the revised manuscript.

Manuscript number: PONE-D-20-35972

Manuscript title: Clinical significance of hypoalbuminemia in patients with scrub typhus complicated by acute kidney injury

**Index of changes**

**Major changes**

1. Description of inclusion criteria in detail in Method section

2. Description of the definition of complication in detail

3. Addition of the reason of hypoalbuminemia in scrub typhus patients in discussion section

4. Addition of the work-up process in Method section

5. Addition of the renal function of AKI group in Table 3

**Minor changes**

1. Change the content of Acknowledgment

Sincerely yours,

Corresponding author: In O Sun, MD., PhD

Division of Nephrology, Department of Internal Medicine

Presbyterian Medical Center, Jeonju, Korea

Phone: +82-2-230-1331, Fax: +82-2-230-1339

E-mail: inogood@catholic.ac.kr

**Responses to Reviewer’s Comments:**
**Reviewer #2:** The authors present a case series of patients diagnosed with scrub typhus admitted to a medical center in South Korea. They demonstrated that hypoalbuminemia detected on presentation was associated with acute kidney injury, correlated with severity of the AKI by RIFLE criteria, and was assessed with other systemic complications. They further state that multiple clinical and laboratory findings were predictive of AKI by multivariate analysis, which may warrant change to assessment, monitoring, or therapeutic approach.

*1. My assessment of their submission is hampered by duplication of Table 1 in the listed Table 2.*

**Response:** Thank you for your comment. We apologize for the mistake. Originally, table 1 and table 2 are totally different. We have changed the table 1, showing the comparison of baseline characteristics between NA and HA.

**Table 1. Comparison of baseline characteristics between normal and abnormal group**

|  | **NA**  **(n=533)** | **HA**  **(n=78)** | **P-value** |
| --- | --- | --- | --- |
| Age | 62 ± 14 | 73 ± 9 | < 0.001 |
| Male, n(%) | 241 (45) | 30 (39) | NS |
| Duration of hospital stay, days | 6.2 ± 3.1 | 9.6 ± 6.2 | < 0.001 |
| DM, n(%) | 90 (17) | 19 (24) | NS |
| Hypertension, n(%) | 188 (35) | 40 (51) | 0.005 |
| CKD, n(%) | 19 (3) | 3 (4) | NS |
| ICU care, n(%) | 11 (2.1) | 16 (20.5) | < 0.001 |
| Hemoglobin (mg/dl) | 12.8 ± 1.6 | 11.5 ± 1.6 | < 0.001 |
| Complications  Gastrointestinal tract, n (%)  Respiratory system  Cardiovascular system  Central nervous system  Acute kidney injury | 68 (13)  73 (14)  58 (11)  4 (0.8)  96 (18) | 8 (10)  39 (50)  22 (28)  5 (6.4)  45 (58) | NS  < 0.001  < 0.001  0.002  < 0.001 |
| Leukocyte (×10^3^/ mL) | 6.7 ± 4.1 | 10.2 ± 3.8 | < 0.001 |
| Platelet count ( ×10^3^/ mL) | 141± 57 | 122 ± 48 | 0.006 |
| Total bilirubin level | 0.7 ± 0.4 | 0.7 ± 0.7 | NS |
| Serum albumin (mg/dl) | 3.7 ± 0.4 | 2.7 ± 0.2 | < 0.001 |
| Serum ALT (IU/L) | 91 ± 129 | 76 ± 56 | NS |
| Creatinine (mg/dl) | 1.0 ± 0.4 | 1.3 ± 0.8 | < 0.001 |
| eGFR ml/min/1.73m^2^ | 71 ± 24 | 50 ± 25 | < 0.001 |

2. *The introduction discusses other publications addressing scrub typhus, its complications, association with renal failure specifically, and associated with other complications. The strengths of this analysis by comparison included a larger sample size and use of RIFLE criteria to demonstrate correlation of severity of AKI with severity of hypoalbuminemia. It is interesting that the rate of hypoalbuminemia in this study was lower than others. I would explicitly state whether others included inpatients, outpatients, or both as that is an important limitation of this study.*

**Response:** Thank you for your valuable comment. In this study, we only included patients who were admitted to our hospital. We excluded outpatient due to lack of follow-up renal function. Therefore, we described such problems as a limitation in discussion section. In addition, we have described the contents in method section to clarify inclusion and exclusion criteria. You mentioned that the rate of hypoalbuminemia in this study was lower than others. We have described the reason in discussion section in detail.

Page 11, line 13

**“In addition, patients who were not admitted to our hospital were excluded from this study.”**

Page 18, line 24

“**In this study, the incidence of hypoalbuminemia was found to be 12.8%, which is relatively lower than that reported previously (25-69.2%) [14,22,23]. We believe that this finding might be due to referral bias. The incidence of hypoalbuminemia found in previous literature is based on data from tertiary care centers; those data were obtained from severe scrub typhus cases [14,22]. In contrast, our hospital is a secondary care center adjacent to a rural area. Thus, many patients may seek treatment at our hospital while suffering from an early-stage infection that has not yet progressed to a severe stage. Furthermore, some patients showing severe scrub typhus were transferred from our hospital to a tertiary care center. Indeed, the AKI incidence was found to be 23.1% in the present study, whereas Hwang et al. reported that the AKI incidence based on RIFLE criteria among scrub typhus cases in a tertiary care center was 35.9% [23].**”

3. *The section on patient selection should explicitly state whether patients were inpatients, outpatients, or both, and whether they included patients transferred in from another facility. Later in the manuscript, it is stated they excluded outpatients. Also they exclude patients transferred to other facilities, but it would be helpful to state if it is known why those patients were transferred, especially it was to a higher level of care.*

**Response:** Thank you for your valuable comment. We have added the content based on your suggestion in method section.

Page 11, line 16

“Patients who were transferred to another hospital **for a higher level of care, or to be treated nearer to their own homes** during the course of treatment or had concomitant infections like leptospirosis, malaria, or dengue fever were excluded from the study**. In addition, patients who were not admitted to our hospital were excluded from this study.** We also excluded patients who were not followed-up during the complete recovery in their renal function or for at least three months after discharge**. However, we included patients who were transferred from a local hospital after their baseline renal function had been checked.**”

4. *Elevation of cardiac biomarkers was stated to define myocarditis—this is not accurate could be stated more accurately as a marker of such. Respiratory complications included ARDS and various image things, but not respiratory failure or mechanical ventilation.*

**Response:** Thank you for your valuable comment. We have added the content based on your suggestion in method section.

Page 12, line 4

The complications were defined based on the presence of dysfunction of the following organ systems: 1) Cardiovascular system - presence of any of the following: systolic blood pressure <90 mmHg, myocarditis (defined **using the diagnostic criteria for clinically suspected myocarditis presented by the European Society of Cardiology [16]** as ~~an elevated creatine kinase-myocardial band isoenzyme above baseline~~), or abnormal cardiac arrhythmia with no previous history of atrial fibrillation, supraventricular tachycardia, or frequent premature ventricular tachycardia; 2) Respiratory system - **presence of any of the following: acute respiratory failure (defined not only by the incidence of hypoxemia, hypercapnia, or the failure to maintain both within a normal range, but also by the presence of any condition that resulted in the need for mechanical ventilation), pneumonia, pleural effusion, pulmonary edema, focal atelectasis, patchy consolidation on chest radiograph or acute distress syndrome**; ~~presence of acute respiratory distress syndrome, defined as the ratio of partial pressure arterial oxygen and fraction of inspired oxygen (PaO2/FiO2) <200 mmHg, with bilateral interstitial infiltration on chest film, or septal lines, pulmonary congestion, patchy consolidation, focal atelectasis, fissure thickening, pleural effusion, and cardiomegaly on chest radiograph;~~

5) Table 3 should include definition of terms by cutoff values. FENa has a marker for footnote ‘a’ but no associated footnote and n(%) appear incorrect. If a larger number of cases did not have FENa or urinalysis available, I question the value of including that information. Was it assessed whether these cases are representative of the population?

**Response:** Thank you for your good suggestion. We apologize for missing the description of footnote ‘a’. We have added the footnote in Table 3. The FENa data are available in 83 patients out of 141 AKI subjects. Although FENa data are not available in all 141 patients with AKI, we believe that showing the data seems to be worthwhile.

Page 17, line 5

**FENa: fractional excretion of sodium**

**^a^ FENa was available in 83 patients.**

6) A bigger question is whether HA as an independent biomarker of AKI risk is significant in this setting. It is correlated with AKI and other poor outcomes, but as a negative acute phase reactant, one would expect that to be true. The homeostasis of albumin is affected by numerous factors other than vascular permeability. During acute inflammatory syndromes, synthesis is suppressed and catabolism increased in addition to increased volume of distribution. The “central mechanism” comment is incomplete. I would direct to the discussion cited as a reference in Lee, et al. (BMC infect Dis 2010).

**Response:** This is a great question. We have changed the discussion section according to your opinion.

Page 18, line 32

“**The occurrence of hypoalbuminemia in patients with scrub typhus seems to be multifactorial; poor dietary intake, decreased synthesis of albumin due to hepatic dysfunction, increased catabolism of protein, and proteinuria are all known to be related to hypoalbuminemia in cases of acute infectious disease [11,14]. The central pathophysiological change associated with scrub typhus is vasculitis and perivasculitis of multiple organs due to the destruction of the endothelial cell lining of small blood vessels and perivascular inflammatory cell infiltration [24-26]. This consequently results in increased vascular permeability with extravascular protein loss. Such a pathomechanism might also contribute to the development of hypoalbuminemia in patients with scrub typhus**.”

7) The fact that almost all cases with AKI were present on admission makes HA a marker for a condition that was already present on admission, not a predictor that it will occur. Does HA predict which cases with AKI will progress to a more significant degree of AKI, or which cases that present with HA and normal renal function will progress to AKI? Would it predict which of the 2% of cases will not recover?

**Response:** Thank you for this important comment. Almost cases recovered their renal function within 72 hours after admission. However, the renal function on admission was not always the worst in this study. The RIFLE class was determined based on the worst renal function in our study, not renal function on admission. Thirty-two of 141 patients with AKI had a worst renal function on the next day after admission in our study. Therefore, we believe that serum albumin level is helpful to predict the development and severity of scrub typhus-associated AKI. To avoid confusion, we have added the renal function at the time of admission, lowest and recovery during admission in table 3.

**Table 3. Clinical characteristics of 141 patients with AKI**

| **Non-oliguric** | 15 (11) |
| --- | --- |
| **FENa < 1%, n (%)^a^** | 53 (64) |
| **Recovery of renal function within 72 h, n (%)** | 138 (98) |
| **Renal function**  **eGFR adm, ml/min/1.73m^2^**  **eGFR low, ml/min/1.73m^2^**  **eGFR rec, ml/min/1.73m^2^** | **39 ± 17**  **36 ± 16**  **74 ± 21** |
| **Urinalysis results**  Proteinuria  Pyuria  Hematuria  normal | 15 (32)  5 (10)  1 (2)  26 (56) |

FENa: fractional excretion of sodium

^a^ FENa was available in 83 patients.

eGFR adm, ml/min/1.73m^2:^ eGFR at the time of admission

eGFR low, ml/min/1.73m^2:^ eGFR at lowest value in hospital

eGFR rec, ml/min/1.73m^2:^ eGFR at the time of recovery

**Reviewer #3:** The authors must be complemented on writing about this very important infectious disease. I had a few queries:

1. *In page 11, could you elaborate further and explain how definitions for cardiac and respiratory complications were arrived at*

**Response:** Thank you for your valuable comment. We have added the content based on your suggestion in method section.

Page 12, line 4

The complications were defined based on the presence of dysfunction of the following organ systems: 1) Cardiovascular system - presence of any of the following: systolic blood pressure <90 mmHg, myocarditis (defined **using the diagnostic criteria for clinically suspected myocarditis presented by the European Society of Cardiology [16]** as ~~an elevated creatine kinase-myocardial band isoenzyme above baseline~~), or abnormal cardiac arrhythmia with no previous history of atrial fibrillation, supraventricular tachycardia, or frequent premature ventricular tachycardia; 2) Respiratory system - **presence of any of the following: acute respiratory failure (defined not only by the incidence of hypoxemia, hypercapnia, or the failure to maintain both within a normal range, but also by the presence of any condition that resulted in the need for mechanical ventilation), pneumonia, pleural effusion, pulmonary edema, focal atelectasis, patchy consolidation on chest radiograph or acute distress syndrome**; ~~presence of acute respiratory distress syndrome, defined as the ratio of partial pressure arterial oxygen and fraction of inspired oxygen (PaO2/FiO2) <200 mmHg, with bilateral interstitial infiltration on chest film, or septal lines, pulmonary congestion, patchy consolidation, focal atelectasis, fissure thickening, pleural effusion, and cardiomegaly on chest radiograph;~~

2. *How was meningoencephalitis identified?*

**Response:** Thank you for your comment. In this study, patients were classified as suffering from meningoencephalitis when they had, in addition to findings of meningitis, abnormal findings on electroencephalograph and/or focal neurologic symptoms and/or altered consciousness without evident cause such as shock or hypoglycemia. Meningitis was defined by the presence of headache or nuchal rigidity with either altered sensorium or focal neurological deficit on history or examination and with cerebrospinal fluid cell counts ≥ 5 leukocytes/mm^3^.

Page 12, line 15

3) Central nervous system - presence of any of the following: Glasgow Coma Scale ≤12 in the absence of any underlying causes, seizure without underlying causes, **meningitis (defined by the presence of headache or nuchal rigidity with either altered sensorium or focal neurological deficits on history or examination, and with cerebrospinal fluid cell counts ≥ 5 leukocytes/mm^3^)**;

*3. Under discussion: “The development of HA in patients with scrub typhus seems to be due to the central pathomechanism of scrub typhus, which is vasculitis or perivasculitis of multiple organs resulting in increased vascular permeability” could you please provide a reference?*

**Response:** Thank you for your comments. We have described the content in detail, and added the reference based on your suggestion.

Page 18, line 32

“**The occurrence of hypoalbuminemia in patients with scrub typhus seems to be multifactorial; poor dietary intake, decreased synthesis of albumin due to hepatic dysfunction, increased catabolism of protein, and proteinuria are all known to be related to hypoalbuminemia in cases of acute infectious disease [11,14]. The central pathophysiological change associated with scrub typhus is vasculitis and perivasculitis of multiple organs due to the destruction of the endothelial cell lining of small blood vessels and perivascular inflammatory cell infiltration [24-26]. This consequently results in increased vascular permeability with extravascular protein loss. Such a pathomechanism might also contribute to the development of hypoalbuminemia in patients with scrub typhus**.”

**24. Strickman D, Smith CD, Corcoran KD, et al. Pathology of rickettsia tsutsugamushi infection in bandicota savilei, a natural host in Thailand. *Am J Trop Med Hyg* 1994, 51: 416-423. PMID: 7943567**

**25. Kim SJ, Chung IK, Chung IS, Song DH, Park SH, Kim HS, Lee MH: The clinical significance of upper gastrointestinal endoscopy in gastrointestinal vasculitis related to scrub typhus. *Endoscopy* 2000, 32: 950-955. doi: 10.1055/s-2000-9621 PMID: 11147943**

**26. Seong SY, Choi MS, Kim IS: Orientia tsutsugamushi infection: Overview and immune responses. Microbes Infect 2001, 3: 11-21. doi: 10.1016/s1286-4579(00)01352-6 PMID: 11226850**

*4. Further explanation of who were included in the study is warranted. We would like to know what work up was done for patients.*

**Response:** Thank you for your valuable comment. We have added the content based on your suggestion in method section.

Page 12, line 3

“The patients' baseline demographic, clinical, and laboratory data were reviewed at the time of hospitalization and during the follow-up period. **Detailed clinical histories of all patients were determined and they underwent thorough physical and biochemical examinations. A standard set of investigations including the following were carried out: complete blood counts, liver function and serum creatinine tests, chest radiograph, three peripheral blood smears for malaria, urinalysis (including urea and electrolytes), two blood cultures, and a standard set of febrile serological investigations including an ELISA for the detection of scrub typhus (InBios International Inc., Seattle, WA). To differentiate between endemic zoonoses including leptospirosis and hemorrhagic fever with renal syndrome (HFRS), another serological work-up was performed. Leptospirosis was diagnosed by a ≥ 4-fold rise in indirect immunofluorescent assay (IFA) titer in paired serum samples, or an antibody titer ≥ 1:800 in one serum sample by the microscopic agglutination test. HFRS was diagnosed by a single titer ≥ 1:80 or ≥ 4-fold rise in IFA titer in paired serum samples.”**
